# Temporal Trends and Geographic Disparity in Hypertension Care in China

**DOI:** 10.2188/jea.JE20190029

**Published:** 2020-08-05

**Authors:** Yang Zhao, Brian Oldenburg, Siqi Zhao, Tilahun Nigatu Haregu, Luwen Zhang

**Affiliations:** 1 Melbourne School of Population and Global Health, The University of Melbourne, Victoria, Australia; 2 WHO Collaborating Centre on Implementation Research for Prevention & Control of NCDs, Victoria, Australia; 3 Yantaishan Hospital of Yantai, Yantai, Shandong, China; 4 Yantai Sino-French Friendship Hospital, Yantai, Shandong, China; 5 School of Health Services Management, Southern Medical University, Guangdong, China

**Keywords:** trends, geographic disparities, hypertension care, China

## Abstract

**Background:**

This study examines trends and geographic disparities in the diagnosis, treatment, and control of hypertension in China and investigates the association between regional factors and hypertension care.

**Methods:**

Blood pressure data and data relating to health care for hypertension were used for this study. The data were sourced from baseline and follow-up surveys of the China Health and Retirement Longitudinal Study, which was conducted in 2011, 2013, and 2015. To estimate the geographical disparities in diagnosis, treatment, and control of hypertension, random-effects models were also applied after controlling for sociodemographic characteristics.

**Results:**

Among hypertensive individuals in China, the trends showed decreases in undiagnosed, untreated, and uncontrolled hypertension: 44.1%, 51.6%, and 80.7% in 2011; 40.0%, 47.4%, and 77.8% in 2013; and 31.7%, 38.0%, and 71.4% in 2015, respectively. The number of undiagnosed, untreated, and uncontrolled hypertensive residents living in urban areas in 2015 was more than 10% lower than the number in rural areas and among rural-to-urban immigrant individuals in China. The poorest socio-economic regions across China were 8.5 times more likely to leave their residents undiagnosed, 2.8 times more likely to leave them untreated, and 2.6 times more likely to leave hypertension uncontrolled.

**Conclusions:**

Although China has made impressive progress in addressing regional inequalities in hypertension care over time, it needs to increase its effort to reduce geographic disparities and to provide more effective treatments and higher quality care for patients with hypertension.

## INTRODUCTION

In an era of sustainable development goals, advancing universal health coverage is the centerpiece of health policy in many countries.^1^^,^^2^ Like most countries in the world, China has recently experienced a very rapid epidemiological transition from a predominance of infectious disease to a predominance of chronic non-communicable diseases (NCDs), including cardiovascular diseases, stroke, diabetes, and cancers.^3^ Hypertension has been a leading risk factor for NCDs and disease burden in China.^4^^–^^6^ As an effective response, the government of China launched a new comprehensive health reform initiative in 2009 aimed at increasing population access to health services, reducing the financial burden of illness, and achieving universal health care (UHC).^4^^,^^7^ As one of five major projects comprising the reform, the National Public Health Service Equalisation (PHSE) Program sought to improve health equity and extend access to primary public health services. It included improved services for the identification and management of individuals with hypertension.

Previous cross-sectional studies have reported the extent of diagnosis, treatment, and control of hypertension^8^^–^^12^; however, there are very few longitudinal studies assessing trends in the geographic distribution of hypertension management following the health system reforms in China.^13^^,^^14^ This study examined trends and geographic variations in the diagnosis, treatment, and control of hypertension from 2011 to 2015 in China, based on a national longitudinal dataset. Additionally, the association between geographic correlates and hypertension care was investigated by applying random-effects models that could estimate the effects of those factors that do not change over time in the longitudinal analysis or in panel studies.

## METHODS

### Data source

This study used longitudinal data from three waves of the China Health and Retirement Longitudinal Study (CHARLS), which was conducted in 2011, 2013, and 2015. This study was designed to collect a high quality, nationally representative sample of Chinese residents aged 45 and older, supporting the scientific research on the elderly and on health trends. A detailed description of the survey objectives and methods can be found elsewhere.^15^ The design of CHARLS was based on the Health and Retirement Study (HRS)^16^ and related ageing surveys, such as the English Longitudinal Study of Aging (ELSA)^17^ and the Survey of Health, Aging and Retirement in Europe (SHARE).^18^ Written informed consent was obtained from all participants. CHARLS received ethics approval from the Peking University Biomedical Ethics Review Committee (Ref. no. IRB00001052-11015) in 2011.^15^

The CHARLS questionnaire covers the following domains: demographics; health status and functioning; health care and insurance; income and consumption; and a number of important biomarkers, including height, weight, and blood pressure. In CHARLS, each respondent’s systolic blood pressure (SBP) and diastolic blood pressure (DBP) were recorded three times by a trained nurse using a HEM-7112 electronic monitor (OMRON, Tokyo, Japan). The average values for each study participant were calculated but only given to the subjects once the interviews were completed. The interviewees were asked if they had hypertension and whether they were receiving any form of anti-hypertensive treatment.^15^

To ensure sample representativeness, CHARLS’ baseline survey covers 150 counties/districts and 450 villages/urban communities across 28 provinces using multi-stage, stratified probability-proportionate-to-size (PPS) sampling. The survey assigned 23,422 dwelling units to interviewers. After excluding empty or non-resident dwellings, 12,740 dwelling units were age-eligible for CHARLS.^15^ A total of 17,708 individuals in 10,257 households were successfully interviewed in the baseline CHARLS survey. The response rate was 80.5% in all age-eligible households in 2011, 85.8% in 2013, and 82.3% in 2015 among all baseline individuals. Ongoing follow-up surveys were conducted every 2 years.

**Figure 1.**
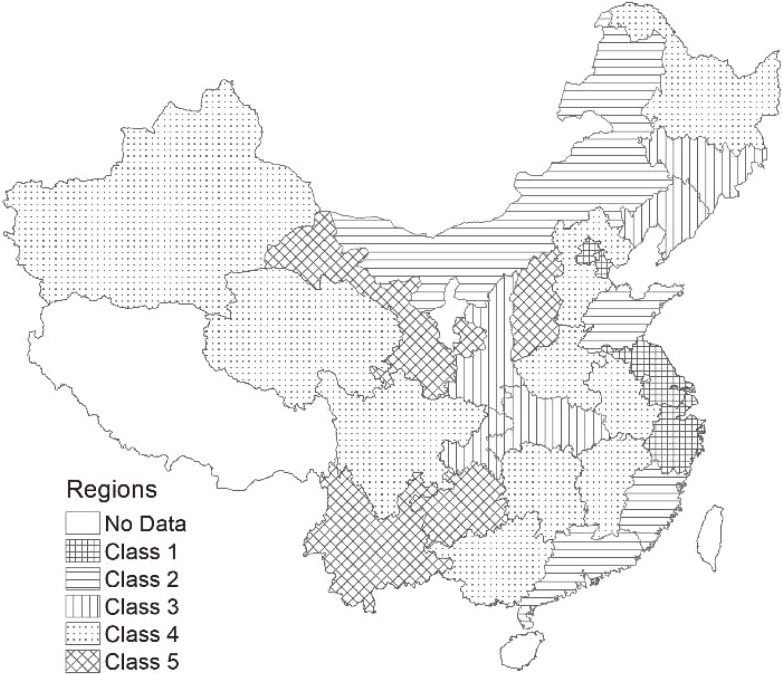
Five classes of the region based on GDP per capita in China in 2015

After excluding cases with missing demographic information and/or blood pressure measurements, complete data were available for 13,725 individuals in 2011, 10,893 individuals in 2013, and 11,675 individuals in 2015. In order to estimate geographical variation in the health care of hypertension, we also created a panel dataset which included 8,486 hypertensive individuals who could be identified in all rounds of the CHARLS. This excluded those unable to be followed-up and those who had died between surveys. In this study, Figure 1 shows that, overall, five classes within the region were identified and ranked, based on their Gross Domestic Product (GDP) per capita at the province level in China: Class 1, >12,000 United States dollar (USD); Class 2, 12,000–10,000 USD; Class 3, 10,000–7,000 USD; Class 4, 7,000–6,000 USD; and Class 5, <6,000 USD.

### Statistical analysis

The prevalence of hypertension, and the rates of undiagnosed, untreated, and uncontrolled hypertension among hypertensive individuals were calculated. In this study, hypertension was defined as systolic blood pressure ⩾140 mm Hg and/or diastolic blood pressure ⩾90 mm Hg and/or reported taking of medication for elevated blood pressure.^19^ Undiagnosed hypertension was defined as those who were found to be hypertensive at the time of physical examination but denied a previous doctor’s diagnosis of hypertension. Untreated hypertension was defined as those with hypertension who had declined anti-hypertensive medication or treatment for high blood pressure over the past year. Uncontrolled hypertension was defined as those with hypertension whose examinations did not have mean systolic blood pressure <140 mm Hg and diastolic blood pressure <90 mm Hg.

The variations in overall prevalence and in rates of undiagnosed, untreated, and uncontrolled hypertension across residential locations and regions of different socio-economic status were analyzed using the Chi-square test. The changes in prevalence, diagnosis, treatment, and control of hypertension across 5 years in each of the sampled 28 provinces were shown in color in geographic information system maps to clearly demonstrate those changes.

In terms of the multivariable analysis, random-effects logistic regression models were also applied to examine the geographical variations in hypertension care, after controlling for demographic characteristics. The explanatory variables of interest were different per capita GDP levels; location of residence (urban area, rural area, or rural-to-urban areas, which was defined as people with agricultural household registration currently living in urban areas); and personal variables, including age, gender, marital status, education, body mass index (BMI), comorbidity, per capita household consumption expenditure, and health insurance. The adjusted odds ratio (AOR) was reported in this study. All statistical analyses were conducted using STATA 14.0 (Stata Corp, College Station, TX, USA). *P* values less than 0.05 were considered statistically significant.

## RESULTS

### Trends in hypertension prevalence and health care in China

Table 1 shows the disparities of hypertension prevalence and management between rural and urban areas. The prevalence of hypertension among Chinese adults aged 45 years and above was around 40% in 2011, 2013, and 2015. Among hypertensive individuals, there were downward trends in undiagnosed, untreated, and uncontrolled hypertension over the study years, with rates of 44.1%, 51.6%, and 80.7% in 2011; 40.0%, 47.4%, and 77.8% in 2013; and 31.7%, 38.0%, and 71.4% in 2015, respectively. The overall level of uncontrolled hypertension was still very high (above 70%) in China in 2015. The prevalence for urban residents was higher than for rural residents during the period 2011 to 2015. For hypertension management, however, the number of undiagnosed, untreated, and uncontrolled hypertensive residents living in urban areas in 2015 was more than 10% lower than the number of patients in rural areas and among rural-to-urban immigrant individuals with hypertension in China (Table 1).

**Table 1.** Hypertension prevalence and health care among Chinese adults from 2011 to 2015, by place of residence

	2011				2013				2015			

	*N*	Unweighted	Weighted	*P* value	*N*	Unweighted	Weighted	*P* value	*N*	Unweighted	Weighted	*P* value
Sample size	13,725	—	—		10,893	—	—		11,675	—	—	
Prevalence												
Urban	1,070	45.6	46.3	0.012	911	48.9	50.1	0.009	828	45.8	45.0	0.053
Rural	3,122	36.1	36.9		2,889	41.0	41.0		3,104	40.5	40.2	
Rural-to-urban	1,093	40.0	43.1		866	43.5	40.4		942	42.8	42.5	
All	5,285	38.5	40.8		4,666	42.8	43.3		4,874	41.8	41.8	
Undiagnosed												
Urban	354	33.1	35.3	0.005	306	33.6	35.9	0.216	205	24.8	23.8	<0.001
Rural	1,439	46.1	46.2		1,133	39.2	39.9		1,036	33.4	34.1	
Rural-to-urban	516	47.2	50.7		351	40.5	46.5		317	33.7	35.3	
All	2,309	43.7	44.1		1,790	38.4	40.0		1,558	32.0	31.7	
Untreated												
Urban	411	38.4	42.0	0.003	375	41.2	42.4	0.114	264	31.9	29.9	<0.001
Rural	1,685	54.0	53.6		1,382	47.8	48.0		1,239	39.9	40.6	
Rural-to-urban	610	55.8	59.2		424	49.0	53.8		391	41.5	41.7	
All	2,706	51.2	51.6		2,181	46.7	47.4		1,894	38.9	38.0	
Uncontrolled	1				1				1			
Urban	773	72.2	75.6	0.003	685	75.2	77.0	0.504	537	64.9	63.6	<0.001
Rural	2,529	81.0	80.6		2,234	77.3	77.0		2,299	74.1	73.8	
Rural-to-urban	911	83.4	86.7		683	78.9	81.1		712	75.6	75.1	
All	4,213	79.7	80.7		3,602	77.2	77.8		3,548	72.8	71.4	

Table 2 shows the disparities between hypertension prevalence and its management in regions of different socio-economic status. The hypertension prevalence was not statistically different across five regions ranked by GDP per capita in China, but the undiagnosed and untreated rate was significantly higher in the regions with lower GDP than in those with higher GDP over the 5 years (weighted undiagnosed: 50.9% vs 14.4%, *P* < 0.01 in 2011, 42.3% vs 12.6%, *P* = 0.017 in 2013; weighted untreated, 60.2% vs 21.0%, *P* < 0.001 in 2011, 52.1% vs 22.3%, *P* = 0.03 in 2013; 41.1% vs 11.7%, *P* = 0.014 in 2015). This study also showed a high rate of uncontrolled hypertension, over 59.5% in regions with the highest GDP and 74.4% in regions with the lowest GDP in 2015, indicating an urgent need for an effective hypertension management plan.

**Table 2.** Prevalence, undiagnosed, untreated and uncontrolled hypertension in China from 2011 to 2015, by region based on economic status^a^

	2011				2013				2015			

	*N*	Unweighted	Weighted	*P* value	*N*	Unweighted	Weighted	*P* value	*N*	Unweighted	Weighted	*P* value
Prevalence											—	
Class 1	120	46.7	54.3	0.052	69	51.9	56.0	0.105	58	53.7	52.1	0.266
Class 2	1,614	40.6	43.2		1,420	43.7	41.5		1,519	43.4	41.5	
Class 3	702	36.8	40.9		604	42.1	43.9		601	40.3	42.2	
Class 4	2,052	36.7	37.5		1,929	42.8	43.8		1,966	40.9	41.6	
Class 5	797	39.9	40.8		644	41.1	42.9		730	41.2	41.3	
Undiagnosed												
Class 1	24	20.0	14.4	<0.001	18	26.1	12.6	0.017	9	15.5	11.1	0.068
Class 2	707	43.8	47.7		528	37.2	42.2		507	33.4	33.4	
Class 3	306	43.6	40.0		248	41.1	38.4		193	32.1	33.1	
Class 4	867	42.3	42.3		744	38.6	40.4		596	30.3	30.2	
Class 5	405	50.8	50.9		252	39.1	42.3		253	34.7	34.8	
Untreated												
Class 1	31	25.8	21.0	<0.001	21	30.4	22.3	0.030	10	17.2	11.7	0.014
Class 2	801	49.6	53.7		640	45.1	48.5		628	41.3	40.7	
Class 3	357	50.9	46.4		302	50.0	47.2		227	37.8	39.2	
Class 4	1,044	50.9	51.0		899	46.6	47.3		729	37.1	36.2	
Class 5	473	59.4	60.2		319	49.5	52.1		300	41.1	41.1	
Uncontrolled												
Class 1	75	62.5	75.0	0.260	43	62.3	56.2	0.062	38	65.5	59.5	0.335
Class 2	1,274	78.9	82.1		1,079	76.0	79.3		1,112	73.2	71.3	
Class 3	575	81.9	79.1		487	80.6	79.9		440	73.2	72.6	
Class 4	1,617	78.8	78.4		1,480	76.7	76.4		1,416	72.0	71.0	
Class 5	672	84.3	85.1		513	79.7	81.9		542	74.3	74.4	

^a^Class 1 is the richest region and Class 5 the poorest region.

**Figure 2.**
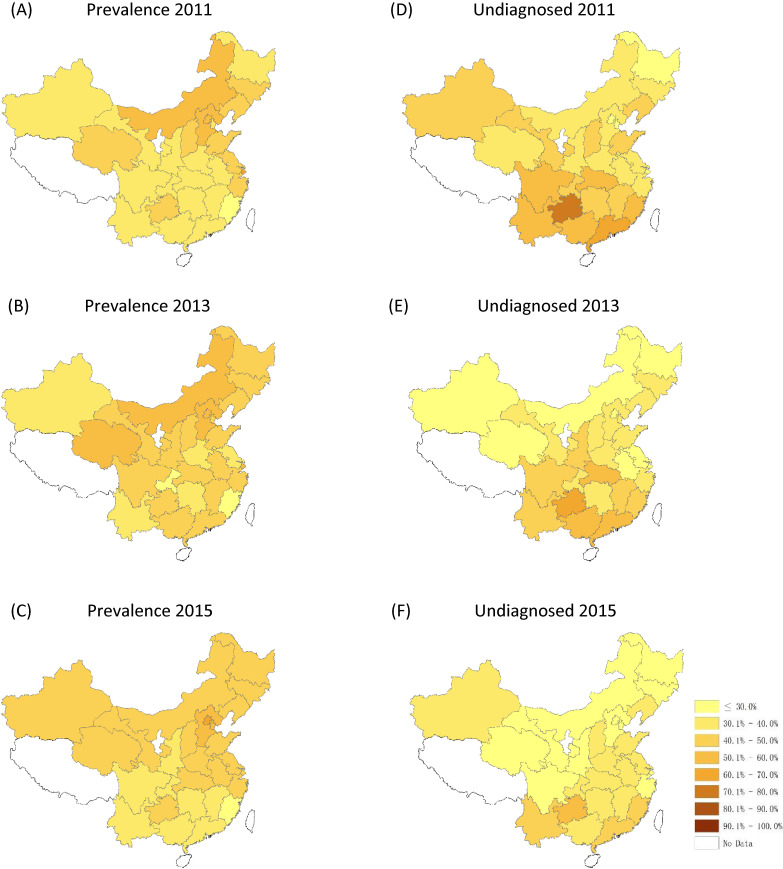
Geographic variation in hypertension prevalence and diagnosis in 2011, 2013, and 2015

**Figure 3.**
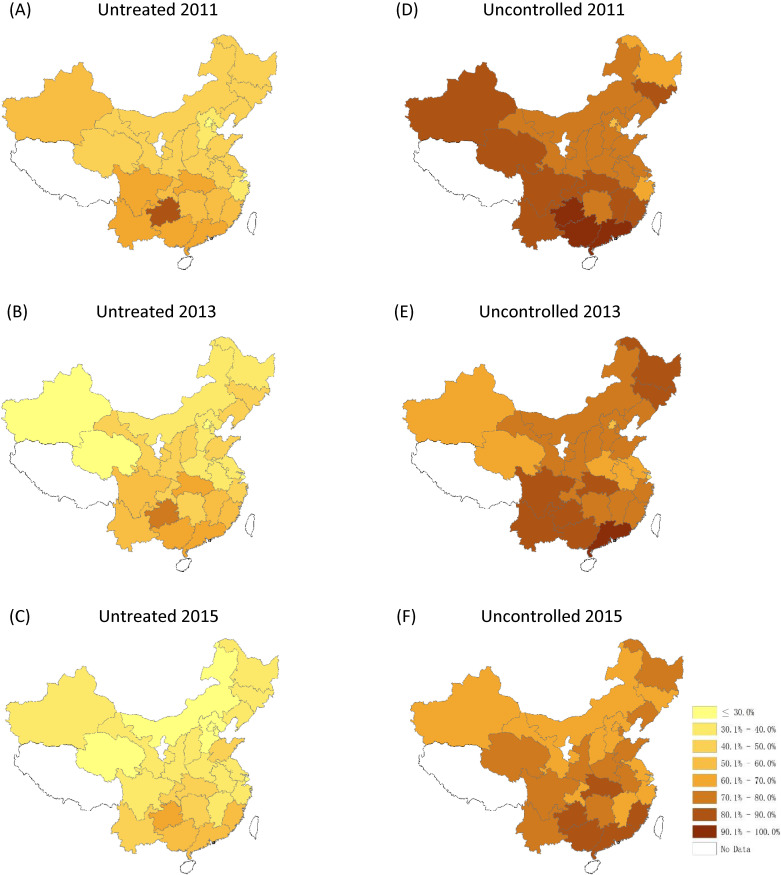
Geographic variation in treatment and control of hypertension in 2011, 2013, and 2015

Figure 2 and Figure 3 show the change in prevalence, and in rates of undiagnosed, untreated, and uncontrolled hypertension in all 28 sampled provinces across China. Significant geographic variations in hypertension management were found between south/north and east/west. Generally, the provinces located in northern China showed a high prevalence of hypertension, while the rates of undiagnosed, untreated, and uncontrolled hypertension were higher in south-western, north-western, and north-eastern areas. Decreasing trends were noted in undiagnosed, untreated, and uncontrolled rates of hypertension at the provincial level in China between 2011 and 2015.

Random-effects models confirmed variations in hypertension diagnosis, treatment, and control across regions with different GDP levels in China. The Class 1 region (with the highest GDP) reported much lower rates of undiagnosed, untreated, and uncontrolled hypertension compared to the other four classes. Areas with the lowest socio-economic indicators were 8.5 times more likely to leave the residents undiagnosed, 2.8 times more likely to leave them untreated, and 2.6 times more likely to leave them uncontrolled. Table 3 also shows that age, gender, lower BMI, higher education, higher income, and insurance coverage were protective factors for hypertension diagnosis, treatment, and control.

**Table 3.** The random effects of region development on hypertension care in China from 2011 to 2015

Variable (reference)	Undiagnosed			Untreated			Uncontrolled		

	AOR	95% CI		AOR	95% CI		AOR	95% CI	
Region^a^ (Class 1)									
Class 2	6.975^***^	3.266	14.895	2.384^***^	1.608	3.535	2.228^***^	1.700	2.920
Class 3	6.651^***^	2.838	15.585	2.542^***^	1.652	3.911	2.651^***^	1.962	3.582
Class 4	3.917^***^	1.951	7.866	2.072^***^	1.436	2.988	1.812^***^	1.414	2.320
Class 5	8.489^***^	3.630	19.854	2.799^***^	1.827	4.290	2.559^***^	1.899	3.450
Residence place (ref: urban)									
Rural	1.030	0.541	1.960	1.354	0.972	1.885	1.068	0.840	1.359
Rural-to-urban	1.684	0.813	3.491	2.115^***^	1.448	3.088	1.494^**^	1.133	1.970
Age (ref: 45–60 years)									
60–70	0.247^***^	0.165	0.371	0.467^***^	0.376	0.580	0.868	0.740	1.017
70–80	0.198^***^	0.114	0.345	0.456^***^	0.341	0.610	1.272^*^	1.024	1.580
≥80	0.106^***^	0.041	0.275	0.487^**^	0.296	0.804	1.420	0.948	2.125
Gender (ref: male)	0.390^***^	0.249	0.612	0.567^***^	0.454	0.707	0.777^**^	0.664	0.908
BMI	0.666^***^	0.631	0.704	0.818^***^	0.796	0.841	0.951^***^	0.934	0.967
Martial status (ref: married)	1.361	0.786	2.357	1.183	0.893	1.569	1.237	0.998	1.534
Education (ref: illiterate)									
Primary school	0.819	0.481	1.395	0.903	0.696	1.170	0.752^**^	0.623	0.908
Middle school & above	0.517^**^	0.276	0.969	0.785	0.574	1.073	0.731^**^	0.584	0.915
Comorbidity (no)	0.040^***^	0.025	0.064	0.234^***^	0.188	0.293	0.437^***^	0.370	0.515
PCE, (ref: <10,000 RMB)	0.357^***^	0.261	0.490	0.573^***^	0.476	0.689	0.717^***^	0.622	0.827
Health insurance^b^ (ref: none)									
UEBMI	0.321^**^	0.139	0.737	0.713	0.444	1.144	0.611^*^	0.420	0.890
URBMI	0.212^**^	0.087	0.517	0.635	0.383	1.055	0.619^*^	0.413	0.928
NCMS	0.471^*^	0.255	0.869	0.647^*^	0.451	0.928	0.673^*^	0.494	0.918
Others	0.705	0.239	2.084	0.738	0.388	1.405	0.696	0.416	1.165

AOR, adjusted odds ratio; BMI, body mass index; PCE, per capita household annual consumption expenditure; UEBMI, Urban Employee Basic Medical Insurance; URBMI, Urban Resident Basic Medical Insurance; NCMS, New Rural Cooperative Medical Scheme.

^***^P < 0.001, ^**^P < 0.01, ^*^P < 0.05 significance test.

^a^Class 1 is the richest region and Class 5 the poorest region.

^b^Others Government health care, private medical insurance and so on.

## DISCUSSION

Hypertension has become the most important public health challenge in China. It accounted for 2.043 million deaths in 2010 and for 6.61% of the 3.187 trillion RMB spent on health care in 2013.^20^ The Chinese government has produced a series of resources and launched a number of intervention programs to deliver affordable, standard hypertensive care in an attempt to stem the spread of hypertension since the 2009 healthcare system reform, including the National Essential Public Health Service Equalisation Program (2009), family physician contracted services, mass health education programs (2012), and provision of essential anti-hypertensive drugs with a low co-payment in primary care facilities (2011). Our research has found an encouraging improvement in hypertension prevalence and in rates of undiagnosed, untreated, and uncontrolled hypertension across urban/rural and regions of different socio-economic status between 2011 and 2015, which is consistent with previous findings^8^^,^^9^^,^^21^^,^^22^ and indicates the efficacy of the current intervention measures taken by the government.^23^^–^^25^

Despite these achievements, huge gaps still exist in the quality of hypertensive management when compared to developed western countries. In Europe, Japan, or North America, for example, more than 80% of hypertensive individuals are aware of their hypertension; more than 80% are receiving anti-hypertensive treatment; and more than 60% have their hypertension well-controlled,^26^^–^^28^ but the rates in China found in this research were only 68.3%, 62.0%, and 28.6%, respectively. This quality gap may be the result of weakness in the service delivery system; the split between preventive care and primary care provision systems may hinder the continuity and coordination of prevention, treatment, and effective control of hypertension.^21^^,^^29^^,^^30^ Unlike hypertensive care patients in most developed countries, those in China are still receiving care from different providers: clinical care is provided by primary care physicians, preventive care and management by public health physicians, community-based intervention by community health workers, and patient education in both primary care facilities and the community.^31^^,^^32^ Efforts in health system strengthening should be focused on bringing the different services together; offering hypertensive patients accessible, continuous, and coordinated care; and improving the quality of hypertension management in China.

Variations in hypertension management were found among individuals in different residential locations. Rural-to-urban migrants reported the highest rates of undiagnosed, untreated, and uncontrolled hypertension; they were 2.115 times less likely to be treated and 1.494 times less likely to be controlled compared to urban residents. The disparities are believed to be caused by the different levels of accessibility and service quality available to the three sub-populations. Migrants face financial obstacles in accessing hypertensive care due to insurance coverage limitations: the New Cooperative Medical Scheme does not cover medical bills outside the pooling units, so when rural-to-urban migrants have hypertensive care requirements outside the pooling units of their insurance, they have out-of-pocket medical costs. In most cases, they choose to neglect their conditions and bear them until they cannot suffer any more.^33^ This postponement of diagnosis and treatment further damages their health, leading to a heavier disease burden.^34^^,^^35^ In addition to such economic constraints, feelings of cultural isolation, low education levels, lack of information, fear of seeing doctors, and malnutrition are all possible factors preventing people from accessing hypertension care or participating in hypertension management programs in urban areas.^36^^,^^37^ Measures were taken to rectify these problems in the majority of population-inflowing cities after 2009, including expanding local health insurance coverage to migrants, offering migrants discounted hypertensive care services in primary care facilities, and strengthening health education and community intervention for migrants.^38^ Chronic condition management for migrants is difficult internationally. More cost-effective technology-related intervention models need to be developed to facilitate screening and follow-up.

Disparities in hypertension management are also found between provinces of different socio-economic status. The Class 1 provinces (with the highest GDP per capita) reported much lower rates of undiagnosed, untreated, and uncontrolled hypertension compared to the other four classes. The random-effects models found that patients in provinces with the lowest socio-economic scores were 8.5 times more likely to be undiagnosed, 2.8 times more likely to be untreated, and 2.6 times more likely to be uncontrolled, indicating the significant effect of socio-economic status on hypertension management. Local governments play a key role in fundraising for almost all hypertension management programs, especially for the National Essential Public Health Service Equalization Program, which offers standardized hypertension management to all patients.^34^ Poorer areas may be less able to successfully fundraise for this purpose. The central government takes responsibility for fundraising for less developed central and western provinces, earmarking central fiscal subsidies for reimbursing 60% and 80% of the cost of their hypertension management programs, respectively, to ensure equitable service quality.^39^ As for Class 1 provinces, the fiscal capacity of their local governments means they can afford various pilot experiments and innovative programs, offer enough incentives to encourage physician teams to promote health education and behavior intervention, and equip facilities with new health management devices and medications, ensuring a higher quality of hypertension services than in other provinces.

Provinces in different regions face different problems; they need to develop specialized HP management plans.^13^ Yin et al divided 31 provinces into five groups based on levels of HP prevalence, awareness, treatment, and control rates. Their results were consistent with our findings.^14^ North and north-western China, in areas such as Hebei, Inner Mongolia, Qinghai, and Gansu, report a higher prevalence of hypertension, with high diagnosis and treatment rates. Lifestyles in the north are a risk factor for high prevalence, with local governments taking active measures to control them successfully.^10^^,^^11^ Some south-eastern provinces, such as Hubei, Fujian, and Guangdong, have lower prevalence, but also have higher rates of undiagnosed, untreated, and uncontrolled hypertension.^12^ We assume that this situation is not caused by local people’s lifestyles but by the presence of a large number of immigrants who have poor management outcomes^40^ because their access to free public services is inadequate. It also reflects the uneven service quality in urban and rural areas.^41^ Some south-western provinces, like Guizhou and Yunnan, report high prevalence and low rates of hypertension diagnosis, treatment, and control. These are under-developed provinces that need more government investment to improve poor health infrastructure, to address low educational levels and unhealthy lifestyles, and to improve chronic disease prevention and control systems.^42^^,^^43^

We also found that age, gender, lower BMI, higher education, higher income, and insurance coverage are protective factors related to hypertension diagnosis, treatment, and control, which is consistent with the previous findings.^11^^,^^44^ Wang et al conducted a national survey with 134,397 residents aged ⩾60 years, finding that the diagnosis and treatment of hypertension was higher in females than in males and higher in urban areas than in rural areas.^41^ Xinglin Feng found that those with insurance that covered the cost of outpatient care were significantly more likely to have their hypertension disorders diagnosed and controlled.^21^ Kjeldsen reviewed recently updated national and international hypertension guidelines, all of which recommended health behavior intervention strategies, including sodium restriction, smoking cessation, body weight reduction, alcohol consumption, and increase in physical activity.^45^ The “2010 Chinese Guideline for the Management of Hypertension” strongly recommended that changed health behaviors should be encouraged to achieve the goals of hypertension control and management.^46^ It is clear that population interventions are needed to control the risk factors in China, with health education and community participation being the most effective measures.

Though this study is one of the first to investigate the temporal and geographic variations in hypertension management in China using nationally-representative longitudinal data, it still has some limitations. CHARLS only includes those aged 45 and older, excluding the younger generation from the analysis. CHARLS was conducted in only 28 provinces, with the data of 6 provinces missing. The average age of the panel would have increased by roughly 2 years in each follow-up survey, since the same respondents were interviewed in all three rounds of the CHARLS survey; this may have had an impact on the estimation of hypertension prevalence. In addition, about 20% of participants did not report their blood pressure readings in all three survey rounds. To account for non-response bias, we adjusted our analysis by using the CHARLS-created weights for individuals, which helped to address this issue.

This study provides an overview of the progress China made towards effective hypertension management between 2011 and 2015. Hypertension prevalence in China stabilized, but disparities in rates of hypertension diagnosis, treatment, and control still exist between rural/urban residents and provinces with of different socio-economic status. China is a large country with large domestic, economic, and regional variations, such that hypertension control cannot be achieved by one intervention plan; it is too complicated. Both a nationally-coordinated service delivery system and province-specific intervention plans are needed to achieve the goal of standardized and high-quality hypertension management.
